# Role of PGC-1α mediated synaptic plasticity, mitochondrial function, and neuroinflammation in the antidepressant effect of Zi-Shui-Qing-Gan-Yin

**DOI:** 10.3389/fneur.2023.1108494

**Published:** 2023-05-12

**Authors:** Wen Zhu, Wen Zhang, Feng Yang, Min Cai, Xiangting Li, Yijin Xiang, Jun Xiang, Yunke Yang, Dingfang Cai

**Affiliations:** ^1^Department of Integrative Medicine, Zhongshan Hospital, Fudan University, Shanghai, China; ^2^Laboratory of Neurology, Institute of Integrative Medicine, Fudan University, Shanghai, China

**Keywords:** depression, Zi-Shui-Qing-Gan-Yin, synaptic plasticity, PGC-1α, neuroinflammation, mitochondrion

## Abstract

Depression is the most prevalent psychiatric disorder, which needs deeper mechanism research studies and effective therapy. Zi-Shui-Qing-Gan-Yin (ZSQGY) is a traditional Chinese medicine decoction that has been widely used in China in the treatment of depressive symptoms. The aim of the study was to examine the anti-depressive effects of ZSQGY and the possible mechanism of action in the monosodium glutamate (MSG)-induced depressive model and the corticosterone (CORT)-induced PC12 cell model. Liquid chromatography-mass spectrometry (LC–MS) was performed to determine the major compounds in the water extract of ZSQGY. The depressive behaviors were evaluated by the field swimming test (FST), the sucrose preference test (SPT), and the open field test (OFT). Golgi staining and transmission electron microscopy (TEM) were performed to display the alterations of synaptic ultrastructure. The mitochondrion function and inflammatory factors were also quantified. The changes in peroxisome proliferator-activated receptor-γ co-activator 1α (PGC-1α) expression were evaluated. The results of this study demonstrated that ZSQGY significantly improved depressive behaviors. ZSQGY also reversed the changes in synaptic plasticity, improved mitochondrion function, and reduced the levels of inflammatory factors. The neuroprotective effects were accompanied by the increased expression of PGC-1α. However, the beneficial changes were reversed after the inhibition of PGC-1α. These results indicated that ZSQGY effectively could improve depressive behaviors *via* the mechanisms that regulate synaptic structural plasticity, improve mitochondrion function, and alleviate neuroinflammation, which could, or partly, attribute to the regulation of PGC-1α.

## Introduction

Depression is a prevalent psychiatric disease with a high recurrence rate and high suicide rate, which has brought heavy health and economic burden to society (1). Approximately 350 million people worldwide suffer from depression, with a lifetime prevalence of 10–20% (2). Among them, approximately 15–25% of the patients eventually died of suicide, accounting for two-thirds of the suicide population (2). The World Health Organization estimates that by 2030, the cost of expanded treatment, mainly psychological counseling and antidepressants, will reach 147 billion dollars (3). It is indisputable that depression has become a significant global public health concern.

Although there are several antidepressant medications, only approximately half of the patients could benefit from the current options and up to 35% of patients are refractory to treatment (4, 5). Severe side effects also make these antidepressant medications far from ideal (6). It is worth noting that the antidepressants currently available are mostly based on the monoamine hypothesis of depression, which cannot explain the delay in therapeutic responses of fast-acting neurotransmitters (7, 8). The imbalance implies that adaptive mechanisms may be involved in the pathogenesis of depression. Therefore, it is necessary to explore the pathogenesis of depression and find more specific and effective therapies.

Decreased synaptic density, mitochondrial dysfunction, and activation of neuroinflammatory factors are closely related to the pathology of depression (9). Peroxisome proliferator-activated receptor-γ co-activator 1α (PGC-1α) is a crucial co-activator that controls gene expression during mitochondrial biogenesis (10). It is also an important coordination factor that participates in the transcriptional regulation of many physiological processes, such as synaptic plasticity and neuroinflammation. The brain is the tissue with high energy demands and extremely depends on mitochondrial function, thus highlighting the crucial role of PGC-1α in the field of neurological disorders (11, 12). Subsequent studies have demonstrated that the expression of PGC-1α is attenuated in depression models and the overexpression of PGC-1α could powerfully improve the synaptic plasticity, mitochondrial function, and inflammatory response (13–15). Considering the role of PGC-1α plays in the physiological and pathological processes, there is a reasonable propose that reduced PGC-1α could contribute to the pathology of depression.

In recent years, increasing studies have reported the efficacy and safety of traditional Chinese medicine (TCM) in relieving symptoms of depression (16, 17). Zi-Shui-Qing-Gan-Yin (ZSQGY) is a TCM decoction that has been widely used in China in the treatment of depressive symptoms for a long history (18). Studies have shown that ZSQGY could alleviate the depressive symptoms of depressed patients and increase the levels of neurotransmitters in the hippocampus of depressed rats (18–21). However, little is known about the mechanisms underlying the antidepressant effects of ZSQGY. The aim of this study was to comprehensively explore the antidepressant effects and mechanism of ZSQGY in the monosodium glutamate (MSG)-induced depressive model and the corticosterone (CORT)-induced PC12 cell model.

## Materials and methods

### Animal

The neonate Sprague–Dawley rats (4–8 g) from Sino-British SIPPR/BK Lab Animals Ltd. (Shanghai, China) were used. The animals were kept under control conditions (12-h light/dark cycle, temperature 23 ± 1°C, relative humidity 55 ± 10%) with free access to water and food. The experiments were performed according to the guidelines of the National Institutes of Health Guide for the Care and Use of Laboratory Animals and approved by the Animal Care and Use Committee of Fudan University. Efforts were made to reduce the number and suffering of animals used in the study.

### Monosodium glutamate (MSG)-induced depressive model

The procedure was performed as previously described (22, 23). MSG (Sigma-Aldrich, St. Louis, MO, United States) was dissolved in 0.9% NaCl solution to the final concentration of 10%. Briefly, the experimental animals were subcutaneously injected with MSG at a dose of 4 mg/g on the postnatal 2nd, 4th, 6th, 8th, and 10th days. The control animals received an equimolar concentration of sodium chloride. The animals were weaned on the 21st day, and the female rats were removed from the cage.

### ZSQGY preparation and quality control

ZSQGY consists of 12 crude drugs including Radix Rehmanniae, Dioscoreae Rhizoma, Radix Bupleuri, Radix Paeoniae Alba, Radix Angelicae Sinensi, Rhizoma Anemarrhenae, Fructus Corni, Cortex Moutan, Poria, Ziziphi Spinosae Semen, Rhizoma Alismatis, and Gardeniae Fructus. The composition of ZSQGY is listed in Table 1. All crude drugs were obtained from Zhongshan Hospital. Briefly, the crude herbs were boiled in 10 times the volume of drinking water for 1 h for three times. Suspensions filtered from three decoctions were mixed and centrifuged at 2,000 *g* for 20 min. The collected suspension was soaked in 100% ethanol under rapid agitation, followed by stirring overnight. Then the suspension was collected and centrifuged at 2,000 *g* for 20 min and concentrated to 2 g/mL (w/v) before autoclaving.

**Table 1:** Composition of ZSQGY.

Ingredients (Latin name)	Ingredients (Chinese name)	Family	Part used	Ratio (g)
Radix Rehmanniae	Di Huang	Scrophulariaceae	Root	12
Dioscoreae Rhizoma	Shan Yao	Dioscoreaceae	Root and rhizome	12
Radix Bupleuri	Chai Hu	Umbelliferae	Root	10
Radix Paeoniae Alba	Bai Shao	Ranunculaceae	Root	10
Radix Angelicae Sinensi	Dang Gui	Umbelliferae	Root	10
Rhizoma Anemarrhenae	Zhi Mu	Liliaceae	Root and rhizome	12
Fructus Corni	Shan Zhu Yu	Cornaceae	Flesh	12
Cortex Moutan	Mu Dan Pi	Ranuculaceae	Velamen	10
Poria	Fu Ling	Polyporaceae	Sclerotium	10
Ziziphi Spinosae Semen	Suan Zao Ren	Rhamnaceae	Seed	15
Rhizoma Alismatis	Ze Xie	Alismatales	Stem	10
Gardeniae Fructus	Zhi Zi	Rubiaceae	Seed	10

### LC–MS analysis of ZSQGY

Liquid chromatography-mass spectrometry (LC–MS) was established for the qualitative analysis of chemical compounds of ZSQGY. The API 4000 tandem mass spectrometer (Applied Biosystems/MDS SCIEX, United States) equipped with an electrospray ionization source (ESI) was used in the LC–MS analysis. All targeted analyses were performed in positive and negative ion modes. The curtain gas (CUR) and collision gas (CAD) used high-purity (99.99%) nitrogen. The ionspray voltage (IS) was set 3,500 V and the ionspray temperature (TEM) was 450°C. The 100 μL samples were dissolved in a 1 mL solution containing methanol and filtered through a 0.22 μm membrane. The content of the components in ZSQGY was calculated using the point external standard method.

### Experimental groups of animals and drug administration

The animals (8 weeks old) were randomly divided into the control group, the model group, the L-ZSQGY group, the M-ZSQGY group, the H-ZSQGY group, and the FXT group. The dose of ZSQGY that translated from human beings to animals in this study was established using the body surface area normalization method. For ZSQGY treatment, the common human daily dose in the present study is 133 g/70 kg bodyweight. According to the formula drat = human × 0.71/0.11, we selected 12, 24, and 48 g/kg/day as low, middle, and high doses, respectively. For fluoxetine treatment, the daily dose required for an adult is 20 mg/70 kg, which is converted to 0.184 mg/100 g for a rat. The treatment dosage for rats was 6.45 times that of patients. The animals in the control group and mode group were taken in the same volume as that of normal saline. All drugs were given once daily continuously for 4 weeks.

### Forced swimming test (FST)

The rats were placed in an acrylic cylinder (height 40 cm, and diameter 20 cm) filled with water (depth 20 cm) at a temperature of 25 ± 1°C for 15 min (the pretest session). After 24 h, the rats were forced to swim again for 5 min. The duration of immobility (s) was analyzed. The rats remained floating in an upright position, with small movements of the four limbs to keep its head above the water surface, and were judged to be immobile.

### Sucrose preference test (SPT)

Briefly, the rats were presented with two bottles containing 1% sucrose solution in the home cage for 24 h. Then, either water or 1% sucrose solution was placed in a random order in the home cage for another 24 h and the bottle order was exchanged every 12 h to account for side preference. The animals were deprived of water for 12 h before the test and then presented with two pre-weighed bottles containing 1% sucrose solution or water. The intake of water, sucrose solution, and total fluid intake were measured after 18 h. The percentage of sucrose solution intake in relation to the total fluid intake was calculated. The impaired sucrose intake was indicative of the depression-like behavior.

### Open field test (OFT)

Central and peripheral areas were divided in the open field apparatus. The animal was placed individually in the open field area (50 × 50 × 50 cm) for 5 min, and the locomotor activity was videotaped (Ethovision 9.0, Noldus). Time spent in center (s) and latency to center (s) were analyzed.

### Golgi staining

Golgi staining and spine density analysis were performed according to the manufacturer’s instructions (Hito Golgi-Cox OptimStain kit, Hitobiotec Corp, Kingsport, TN, United States). The medial prefrontal cortex (mPFC) was cut into 80 μm thickness coronal tissue sections using a freezing microtome (Microm HM 450, Waldorf, Germany). Neurons in the mPFC area were analyzed. Dendritic spine density was counted by randomly selecting the secondary and tertiary apical dendrites and was shown as the number of thorns/10 μm dendrite. In total, three segments were counted per section, and three slides were chosen from each rat.

### Transmission electron microscopy (TEM)

The rats were given anesthesia with sodium pentobarbital (40 mg/kg) intraperitoneal injection, and the mPFC were removed and incubated in 2.5% glutaraldehyde at 4°C for 24 h. Then, the mPFC were cut into 1 mm^3^ segments and fixed in 1% osmium tetroxide for 2 h. The tissue segments were rinsed and dehydrated in a series of graded aqueous ethanol and embedded in Epon. A total of 70 nm ultrathin sections were prepared and stained with 3% uranyl acetate and 0.5% lead citrate. Images were taken using the TEM (6,200× magnification) and analyzed using Image Pro Plus.

### Immunohistochemistry (IHC)

The rats were anesthetized with sodium pentobarbital (40 mg/kg) intraperitoneal injection and then transcardially perfused with 4% paraformaldehyde. The brains were removed and then paraffin was embedded. Then the mPFC slides (10 μm) were incubated in PBS containing 0.03% H_2_O_2_ for 10 min and in 5% goat serum for 30 min, followed by the incubation with anti-PGC-1α (1:100; Abcam, Cambridge, United Kingdom) at 4°C overnight. After washing with PBS, the sections were incubated with a secondary antibody (1:200) for 1 h. Then slices were added an appropriate amount of horseradish enzyme and incubated at 37°C for 30 min. After washing with PBS, the slides were incubated in DAB. The nuclei were stained with hematoxylin. The slices were dehydrated with ethanol, sealed with gum, and further observed under the microscope for observation and analysis (Olympus BX51, Tokyo, Japan).

### qRT-PCR

The total RNA was extracted using a Trizol Reagent (Introgen, Carlsbad, CA, United States) according to the instructions. The total RNA was reverse transcribed into cDNA using the cDNA synthesis kit (Thermo Fisher Scientific, Waltham, MA, United States). A real-time quantitative PCR analyzer was used to detect the expressions of PGC-1α mRNA and PSD95 mRNA. The obtained genes were normalized to GAPDH. The primers sequences were listed as follows: GAPDH, AACTCCCATTCCTCCACCTT, and GAGGGCCTCTCTCTTGCTCT; PGC-1α, AGGCAAGCAAGCAGGTCT, and GTCATCAAACAGGCCATCC; and PSD95, GCAGGTTGCAGATCGGAGAC, and CCAGGTGCTGAGAATATGAGGTT.

### Measurement of mitochondrial DNA (mtDNA)

The analysis of the mtDNA amount was measured by quantitative PCR. The copy numbers of mtDNA were normalized to a nuclear-encoded gene RBM15.

### Enzyme-linked immunosorbent assay

The concentrations of malondialdehyde (MDA), 8-hydroxy-2-deoxyguanosine (8-OHdG), tumor necrosis factor-α (TNF-α), interferon-γ (IFN-γ), interleukin-1 (IL-1), 6 (IL-6) from the mPFC of rats, and cell serum were quantified using enzyme-linked immunosorbent assay (ELISA) kits (Shanghai Enzyme-linked Biotechnology, Shanghai, China).

### Measurement of mitochondrial ATP level

The mitochondrial ATP level was detected using the ATP Assay kit (Beyotime Biotechnology, Shanghai, China) according to the manufacturer’s instructions.

### Preparation of the ZSQGY-containing serum

The rats were administered with the ZSQGY (24 g/kg/day) for 3 consecutive days. After anesthetized with sodium pentobarbital (40 mg/kg), the abdominal aorta blood of the animals was collected 2 h after the treatments and centrifuged at 4°C for 20 min, followed by sterilization through a 0.22 μm microporous membrane. The serum was stored at −80°C.

### Cell culture and drug treatment

High concentrations of CORT can induce the neurotoxicity of PC12 cells, which has been widely used as an *in vitro* model for depression. In this study, differentiated PC12 cells were maintained (at a density of 1 × 105 cells/mL) in RPMI-1640 supplemented with 10% heat-inactivated fetal bovine serum and 1% penicillin–streptomycin at 37°C under a humidified atmosphere of 5% CO_2_ and 95% air (v/v) for 24 h. Plated PC12 cells were exposed to CORT (Sigma-Aldrich, St. Louis, MO, United States) at the level of 200 and 400 μM for 24 h, and then the PC12 cells were treated with different concentrations of the ZSQGY-containing serum (5, 10, and 20%) to determine the suitable doses of CORT and ZSQGY-containing serum by assessing with the CCK-8 kit (Beyotime Biotechnology, Shanghai, China).

### Viability assay

PC12 cells were treated with the CCK-8 solution and incubated at 37°C for 4 h. The optimal density at 450 nm was measured with a microplate reader (Bio-Rad Laboratories, Hercules, CA, United States). Cell viability was presented as a percentage of control cells.

### Grouping of PC12 cells and PGC-1α siRNA transfection

The PC12 cells were divided into the control group, the model group, the ZSQGY + PGC-1α siRNA group, and the ZSQGY + con-siRNA group. For the control group, PC12 cells were cultured under the abovementioned normal conditions. For the model group, PC12 cells were exposed to 200 μM CORT for 24 h. For the ZSQGY + PGC-1α siRNA group, PC12 cells were treated with PGC-1α siRNA before modeling and ZSQGY-containing serum treatment. For the ZSQGY + con-siRNA group, PC12 cells were treated with con-siRNA before modeling and ZSQGY-containing serum treatment. The PGC-1α siRNA and con-siRNA (Jikai Biology, Shanghai, China) were transfected with Lipofectamine 2000 (Invitrogen, Carlsbad, CA, United States).

### Statistical analysis

Data are expressed as mean ± standard error. Data were analyzed by one-way analysis of variance (ANOVA) followed by the LSD multiple comparison test using software SPSS 20.0. Data were considered statistically significant at a value of *p* of less than 0.05.

## Results

### Qualitative and quantitative analysis of components in water extract of ZSQGY

**Figure 1:**
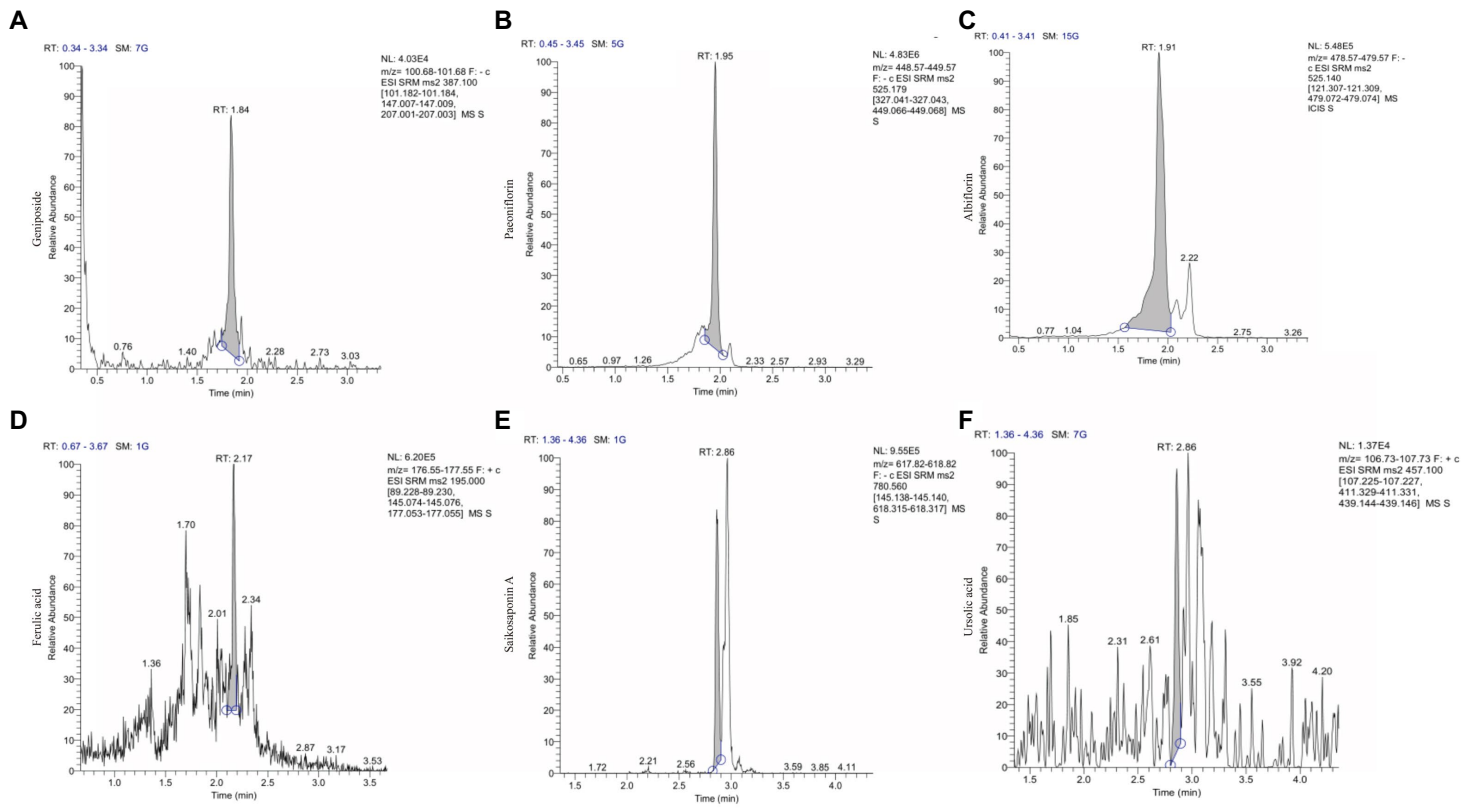
The ion chromatograms (TICs) of geniposide, paeoniflorin, albiflorin, ferulic acid, saikosaponin A and ursolic acid in ZSQGY. **(A)** Geniposide; **(B)** Paeoniflorin; **(C)** Albiflorin; **(D)** Ferulic acid; **(E)** Aaikosaponin A; **(F)** Fursolic acid.

Qualitative analysis of compounds in water extract of ZSQGY was carried out by LC–MS. A total of six compounds, including geniposide, paeoniflorin, albiflorin, ferulic acid, saikosaponin A, and ursolic acid in water extract were used for quantitative analysis (Figure 1). After calculation, this water extract contained 533.682 μg/mL of geniposide, 474.789 μg/mL of paeoniflorin, 225.892 μg/mL of albiflorin, 280.147 μg/mL of ferulic acid, 30.411 μg/mL of saikosaponin A, and 11.468 μg/mL of ursolic acid, respectively (Table 2).

**Table 2:** Quantitative analysis of six major compounds in ZSQGY water extract analyzed by LC–MS.

Chemical ingredients	Liner range (μg/mL)	Regression equation	Correlation coefficient^®^	Actual concentration (μg/mL)
Geniposide	1–100	*Y* = 3221.15 + 9301.68x	0.9933	533.682
Paeoniflorin	0.1–50	*Y* = −28193.1 + 1.18247e+006x	0.9922	474.789
Albiflorin	0.01–100	*Y* = −2638.02 + 65,849x	0.9953	225.892
Ferulic acid	1–100	*Y* = −69442.4 + 193,791x	0.9922	280.147
Saikosaponin A	0.01–10	*Y* = 1507.57 + 2.39811e+006x	0.9911	30.411
Ursolic acid	0.1–10	*Y* = −2287.96 + 136,156x	0.9993	11.468

### ZSQGY improved the depressive behaviors in the MSG-induced depressive model

**Figure 2:**
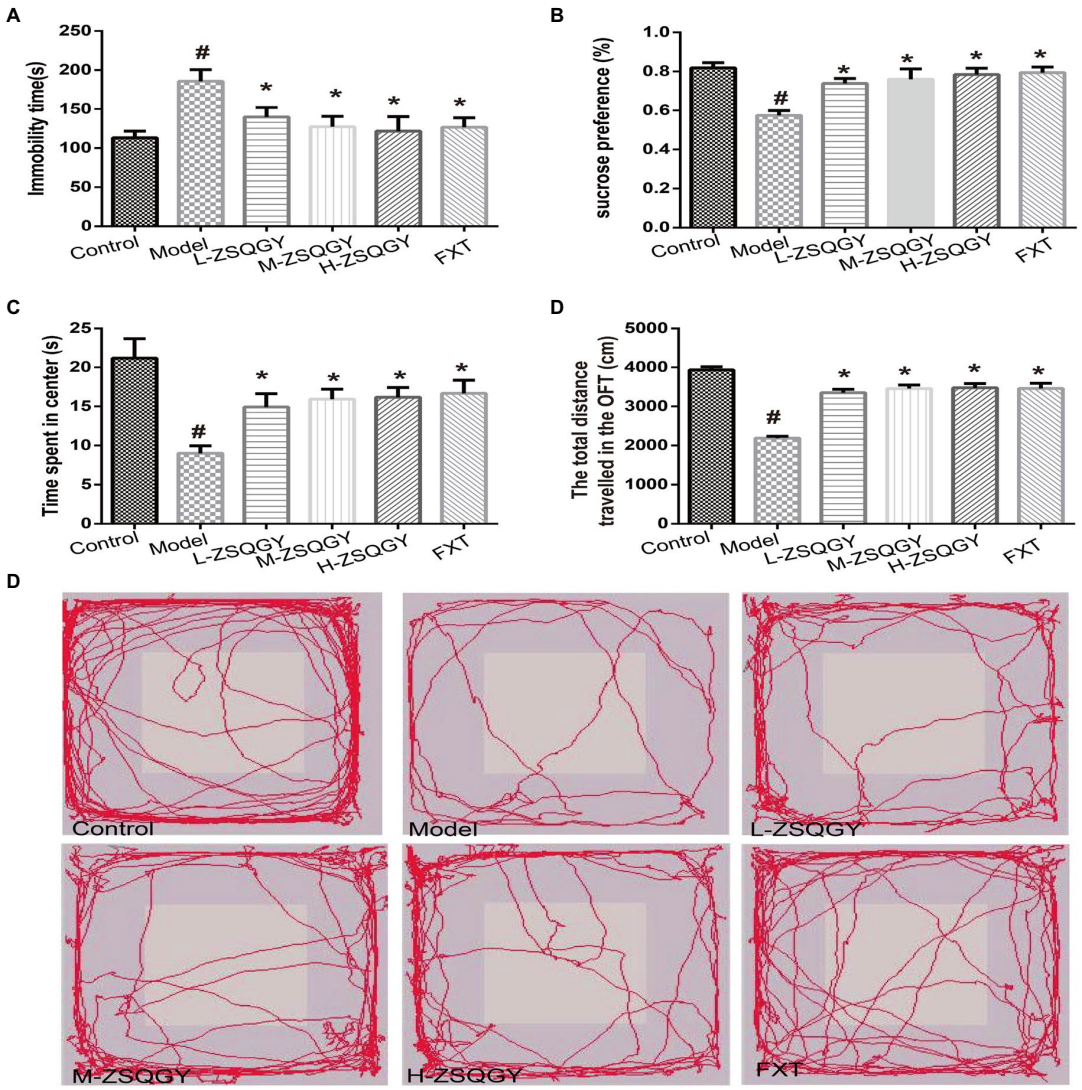
ZSQGY improved depressive behaviors in the MSG-induced depressive model. **(A)** Immobility time in the forced swimming test. **(B)** Sucrose preference (%) in the sucrose preference test. **(C)** Time spent in center (s) in the open field test. **(D)** Total distance traveled in the open field test. **(E)** Trace plot of the open field test. Data are presented as mean ± SE. #*p* < 0.05 vs. control group. *p < 0.05 vs. model group (*n* = 12 in each group).

The forced swimming test, the sucrose preference test, and the open field test were administrated to evaluate the effects of ZSQGY against depressive behaviors. In the forced swimming test, the rats in the model group spent longer immobility time during the test (*p* < 0.05; Figure 2A). The rats in the ZSQGY group and fluoxetine group spent shorter immobility time than the rats in the model group (*p* < 0.05; Figure 2A). In the sucrose preference test, we observed that compared with the control group, the consumption of sucrose in the model group decreased (*p* < 0.05; Figure 2B). However, the consumption of sucrose in rats that received ZSQGY or fluoxetine treatment increased significantly (*p* < 0.05; Figure 2B). In the open field test, the rats in the model group spent less time in the central area than those in the control group (*p* < 0.05; Figures 2C–E). The rats in the model group also traveled a shorter distance than those in the control group. In contrast, the rats who received ZSQGY or fluoxetine treatment spent a longer time in the central area and traveled a longer distance (*p* < 0.05; Figures 2C–E).

### ZSQGY ameliorated impaired synaptic structural plasticity in mPFC in the MSG-induced depressive model

**Figure 3:**
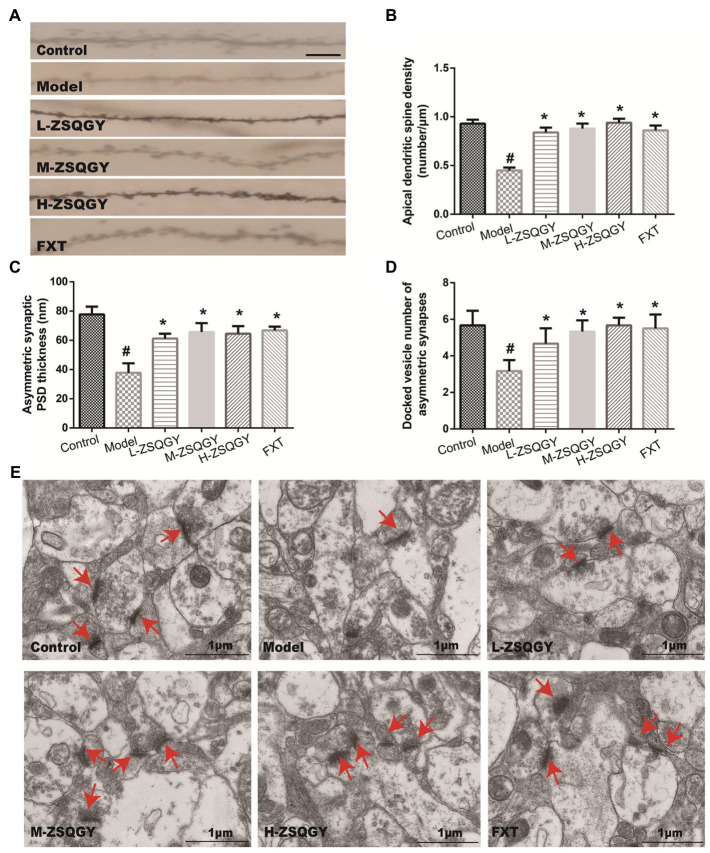
ZSQGY ameliorated impaired synaptic structural plasticity in mPFC in the MSG-induced depressive model. **(A)** Golgi staining of the apical dendritic spine (scale bar, 5 μm). **(B)** Apical dendritic spine density (number/μm). **(C)** Asymmetric synaptic PSD thickness (nm). **(D)** Docked vesicle number of asymmetric synapses. **(E)** Ultrastructure of asymmetric synapses in the mPFC (scale bar, 1 μm). Data are presented as mean ± SE. #*p* < 0.05 vs. control group. *p < 0.05 vs. model group (*n* = 6 in each group).

Results from the Golgi staining showed that compared with the control group, the density of dendritic spines in mPFC in the MSG group induced decreased significantly (*p* < 0.05; Figures 3A,B). The treatments of ZSQGY and FXT significantly reversed a decrease in dendritic spine density induced by MSG (*p* < 0.05; Figures 3A,B). To further corroborate the results from Golgi staining, the mPFC neuronal ultrastructure was examined by TEM. The PSD thickness in asymmetric synapses in the model group was significantly thinner than those in the control group (*p* < 0.05, Figures 3C,E). However, the treatments of ZSQGY and FXT remarkably ameliorated the reduction of the PSD thickness (*p* < 0.05, Figures 3C,E). In addition, the number of docked vesicles in asymmetric synapses was decreased in the model group (*p* < 0.05, Figures 3D,E). However, the reduction was reversed by the treatment of ZSQGY or FXT (*p* < 0.05, Figures 3D,E).

### ZSQGY attenuated the damaged mitochondrial function in the MSG-induced depressive model

**Figure 4:**
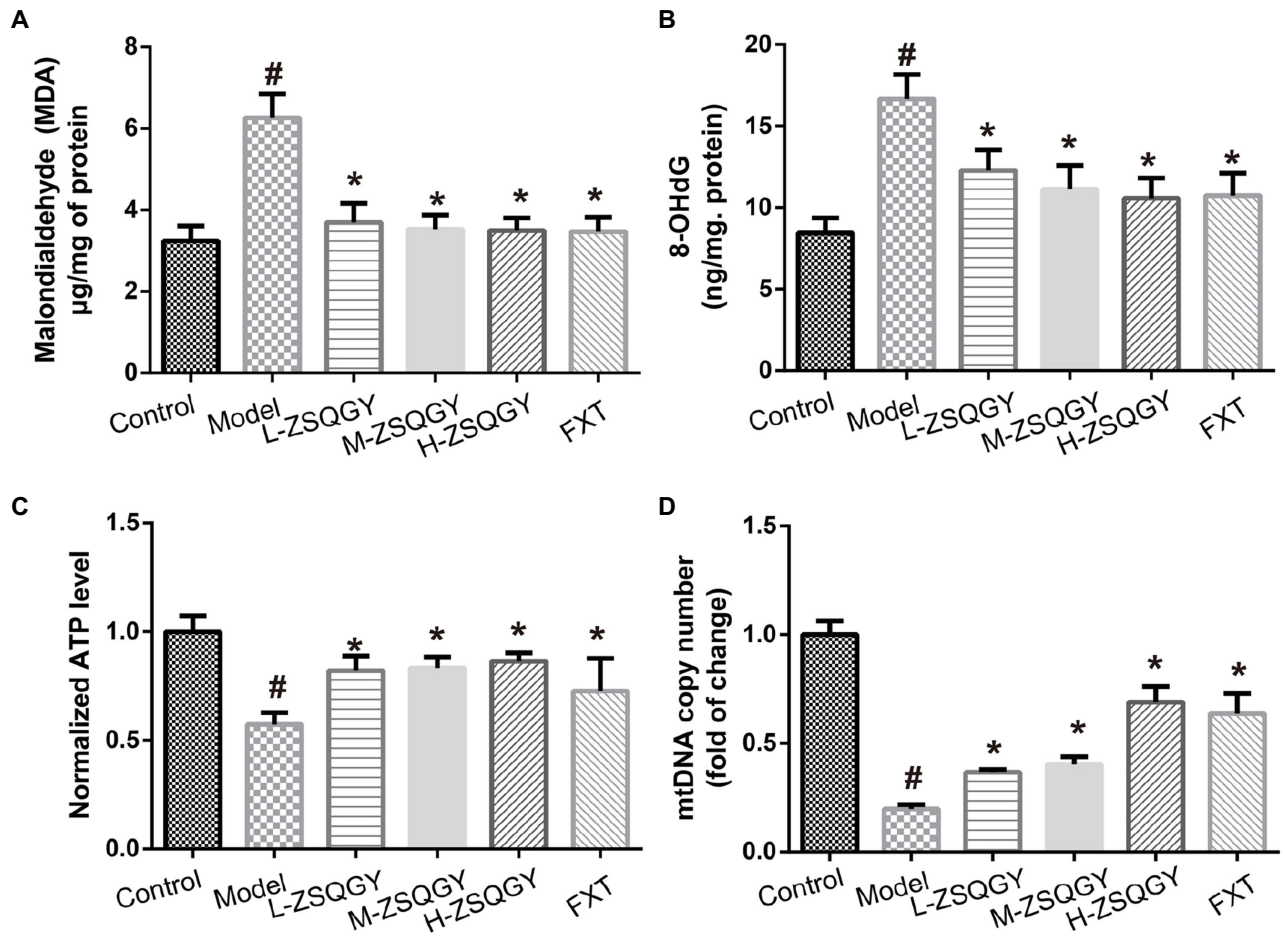
ZSQGY attenuated the damage of mitochondrial function in the MSG-induced depressive model. **(A)** ELISA of MDA. **(B)** ELISA of 8-OHdG. **(C)** ATP content. **(D)** mtDNA copy number. Data are presented as mean ± SE. #*p* < 0.05 vs. control group. *p < 0.05 vs. model group (*n* = 6 in each group).

As shown in Figure 4, the levels of 8-OHdG and MDA in the model group were higher when compared with those in the control group (*p* < 0.05; Figures 4A,B). Contrarily, the levels of 8-OHdG and MDA decreased in rats that received the treatment of ZSQGY or FXT (*p* < 0.05; Figures 4A,B). In addition, the ATP content and mtDNA copy number of the model group were significantly decreased when compared with those in the control group (*p* < 0.05; Figures 4C,D). The application of ZSQGY eminently counteracted the decrease of ATP content and mtDNA copy number (*p* < 0.05; Figures 4C,D).

### ZSQGY attenuated the level of inflammatory cytokines in the MSG-induced depressive model

**Figure 5:**
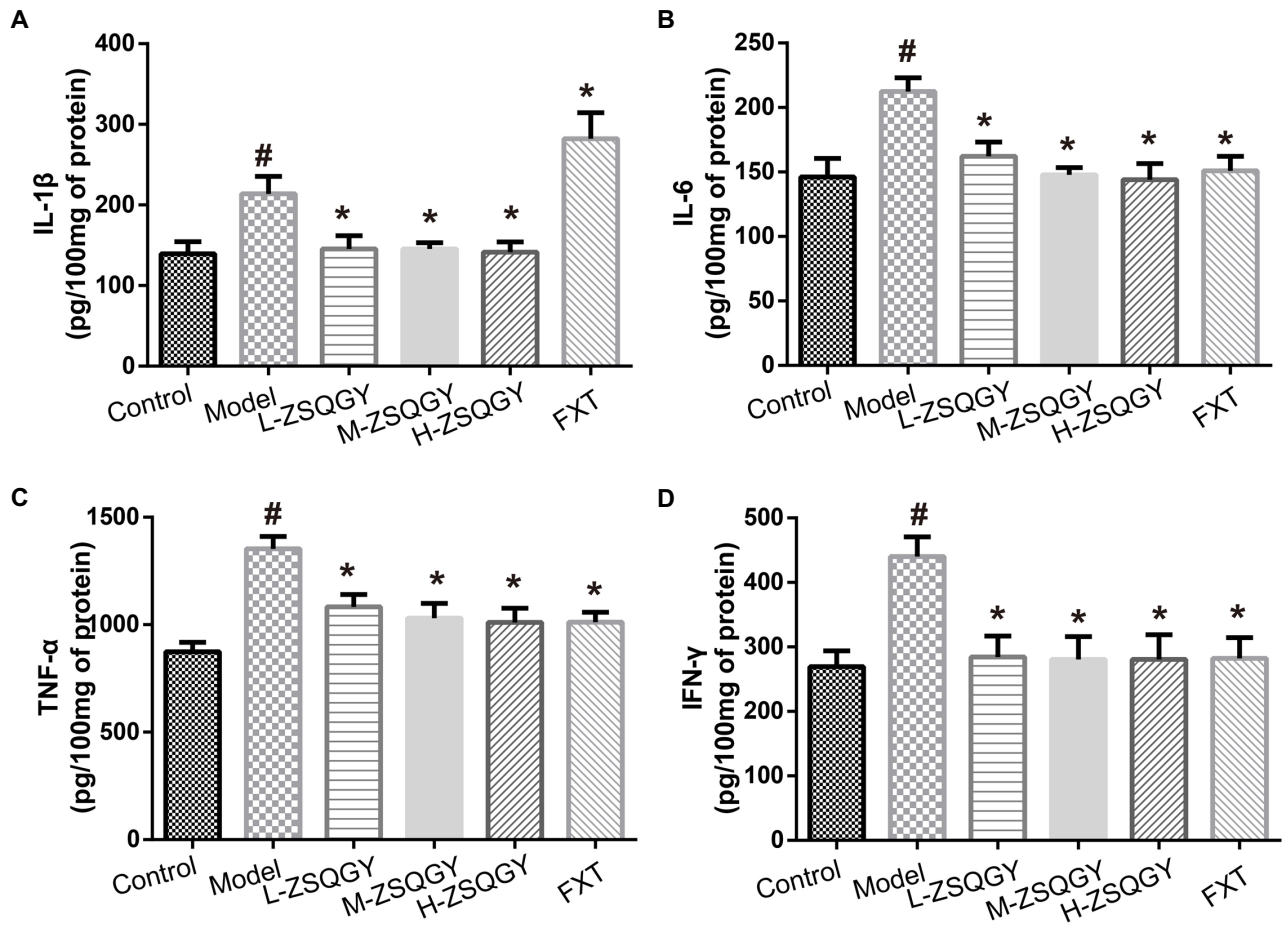
ZSQGY attenuated the level of inflammatory cytokines. **(A)** Level of IL-1β. **(B)** Level of IL-6. **(C)** Level of TNF-α. **(D)** Level of IFN-γ. Data are presented as mean ± SE. #*p* < 0.05 vs. control group. *p < 0.05 vs. model group (*n* = 6 in each group).

As shown in Figure 5, the levels of IL-1β, IL-6, TNF-α, and IFN-γ were elevated in the model group compared to those in the control group (*p* < 0.05; Figures 5A–D). Notably, ZSQGY and FXT reduce the content of inflammatory cytokines induced by MSG (*p* < 0.05; Figures 5A–D).

### ZSQGY attenuated the reduction of PGC-1α in the MSG-induced depressive model

**Figure 6:**
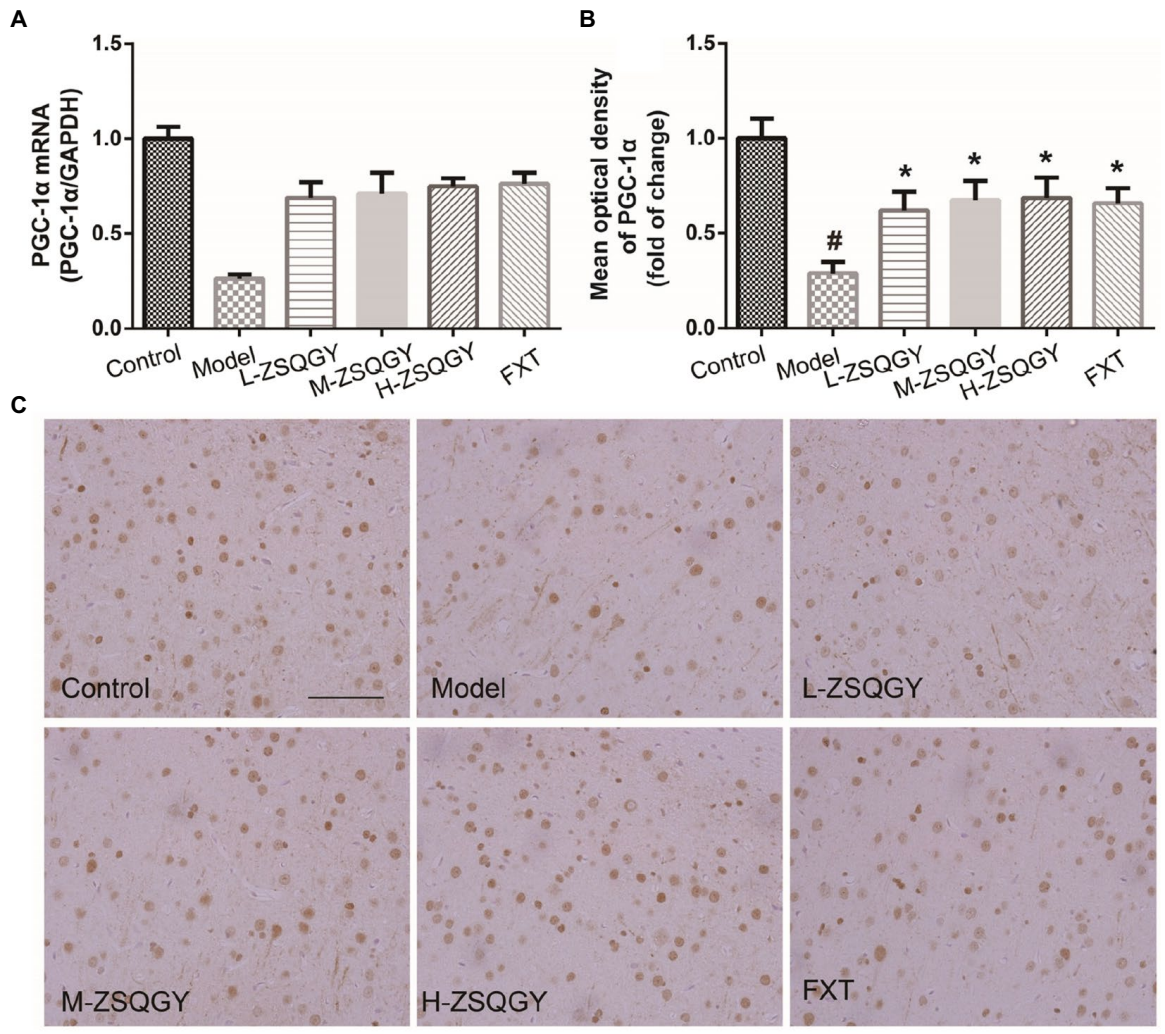
ZSQGY attenuated the reduction of PGC-1α in the MSG-induced depressive model. **(A)** Level of PGC-1α mRNA. **(B)** Mean optical density of PGC-1α. **(C)** IHC of PGC-1α. Data are presented as mean ± SE. #*p* < 0.05 vs. control group. *p < 0.05 vs. model group (*n* = 6 in each group).

As shown in Figure 6, the level of PGC-1α mRNA significantly decreased in the model group compared with those in the control group (*p* < 0.05; Figure 6A). However, the level of PGC-1α mRNA significantly increased in the rats that received ZSQGY or FXT (*p* < 0.05; Figure 6A). The results of IHC showed that PGC-1α is stained brownish yellow or brown and expressed in both the nucleus and cytoplasm. In comparison with the control group, the mean optical density of PGC-1α was significantly decreased (*p* < 0.05; Figures 6A,B). In contrast, the mean optical density of PGC-1α was increased remarkably in the ZSQGY group and in the FXT group (*p* < 0.05; Figures 6A,B).

### ZSQGY protects PC12 cells against CORT-induced injury

**Figure 7:**
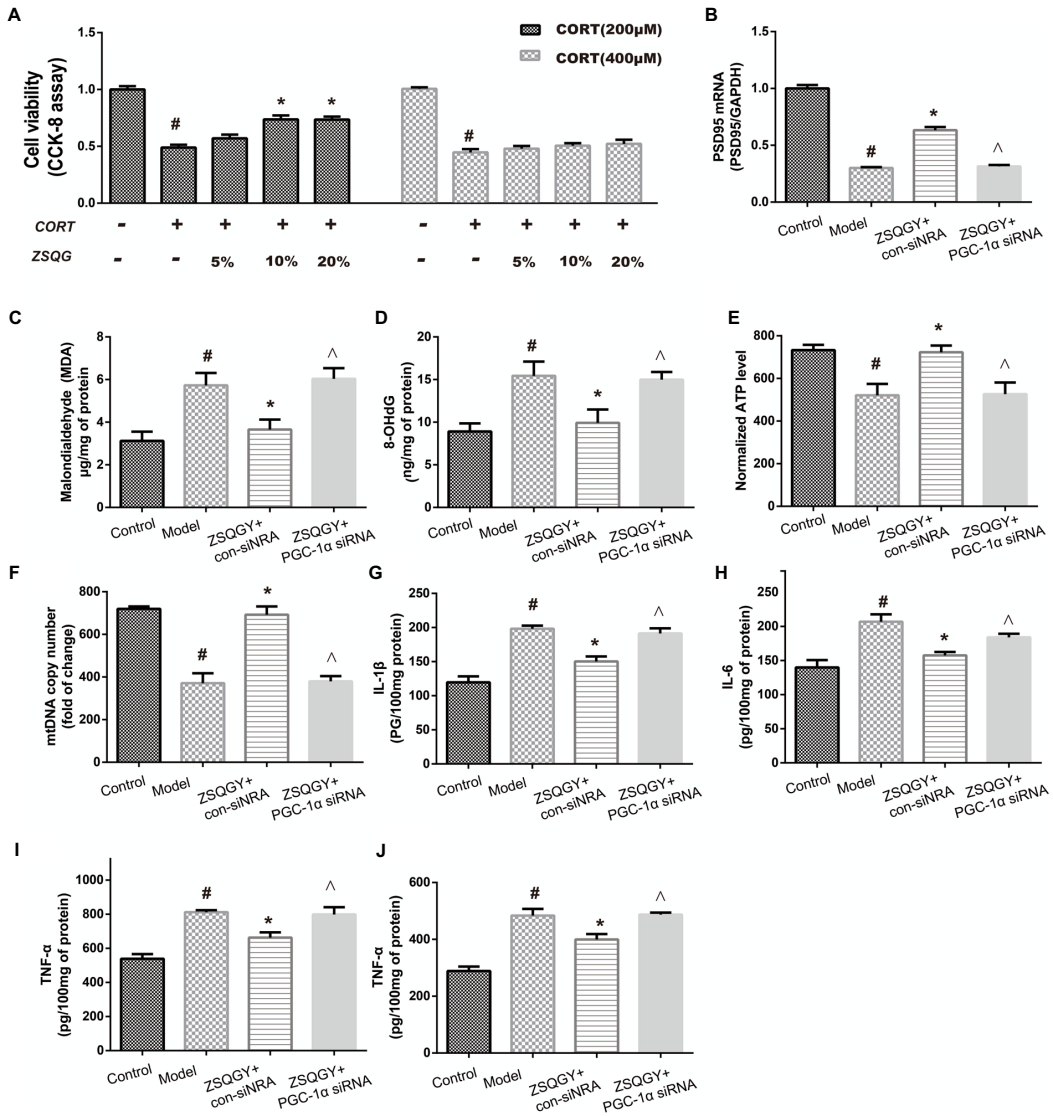
ZSQGY protects PC12 cells against CORT-induced injury. **(A)** Cell activity measured CCK8 assay. **(B)** Level of PSD95 mRNA. **(C)** ELISA of MDA. **(D)** ELISA of 8-OHdG. **(E)** ATP content. **(F)** mtDNA copy number. **(G)** Level of IL-1β. **(H)** Level of IL-6. **(I)** Level of TNF-α. **(J)** Level of IFN-γ. Data are presented as mean ± SE. #*p* < 0.05 vs. control group. *p < 0.05 vs. model group. ^*p* < 0.05 vs. ZSQGY + con-siRNA group (*n* = 6 in each group).

To screen out the appropriate concentration of CORT to damage PC12 cells as well as the cytoprotective effects of ZSQGY against CORT-induced injury, the PC12 cells were treated with high concentrations of CORT (200 and 400 μM) for 24 h, followed by treatment with different concentrations of ZSQGY-containing serum (5, 10, and 20%) for 24 h. As shown in Figure 7A, 200 μM CORT treatment caused a decrease of approximately 50% of viable cells. In addition, 10% ZSQGY-containing serum significantly increased cell viability, while there were no significant changes in the viability of cells in PC-12 cells treated with 5 and 20% ZSQGY-containing serum. Therefore, 200 μM CORT and 10% ZSQGY-containing serum were selected for the following studies.

To further explore whether PGC-1α is implicated in the beneficial effects of ZSQGY, the PGC-1α siRNA was used to inhibit its functions and then the levels of postsynaptic density 95 (PSD95), oxidative stress markers, and inflammatory cytokines were determined. As shown in Figures 7B–J, compared to the control group, the levels of oxidative stress markers (8-OHdG, MDA) and inflammatory cytokines (IL-1β, IL-6, TNF-α, and IFN-γ) were significantly increased in the CORT-induced PC12 cells, while the levels of PSD95 mRNA, ATP, and mtDNA were decreased in the CORT-induced PC12 cells (*p* < 0.05; Figures 7B–J). The ZSQGY-containing serum treatment notably decreased the levels of 8-OHdG, MDA, and inflammatory cytokines (IL-1β, IL-6, TNF-α, and IFN-γ) (*p* < 0.05; Figures 7B–J). This treatment also significantly increased the levels of PSD95 mRNA, ATP, and mtDNA (*p* < 0.05; Figures 7B–J). However, the beneficial effects of ZSQGY in synaptic plasticity, mitochondrial function, and neuroinflammation were blocked after the administration of PGC-1α siRNA (*p* < 0.05; Figures 7B–J).

## Discussion

In this study, we evaluated the antidepressant effects and mechanism of ZSQGY. The results showed that ZSQGY could improve depressive behaviors in the MSG-induced depressive model. In addition, ZSQGY significantly improved the damaged synaptic structural plasticity, attenuated the dysfunction of the mitochondrion, and reduced the level of inflammatory cytokines in *in vivo* and *in vitro* depression models. However, the beneficial changes were reversed after the inhibition of PGC-1α.

One of the important pathological features of patients with depression is the damaged synaptic density, which could lead to the interruption of neural circuits and the abnormality of brain structure caused (24, 25). Studies have reported that a reduced size of the prefrontal cortex (PFC) and decreased neuronal synapses are associated with depression (26). The dendritic spine is the morphological specialization that protrudes from the dendritic shaft. Structural changes in the dendritic spine are considered the basis of synaptic plasticity. Converging evidence from clinical and experimental studies indicates that the impaired dendritic spine is involved in the pathology of multiple diseases (27). The loss of dendritic spines is accompanied by depression-like behaviors, suggesting that the structural changes and neuronal atrophy in mPFC are associated with depression (28–30). Moreover, synaptic ultrastructural alterations, such as decreased PSD thickness, are associated with depressive behavioral changes (31, 32). In this study, ZSQGY remarkably increased the dendritic spine density, the PSD thickness, and the number of docked vesicles, indicating that ZSQGY administration could improve the damaged synaptic structural plasticity.

The energy metabolism disorder caused by mitochondrial dysfunction contributes to the pathogenesis of depression (33). As the energy factory of cells, mitochondria participates in maintaining calcium homeostasis and regulating the generation of reactive oxygen species (ROS) and cell apoptosis (34). Moreover, mitochondria in the central nervous system can enable synaptic plasticity and promote neural differentiation and neurotransmitter release (35). Internal and external cues can lead to the reduction of ATP synthesis and excessive production of ROS, which may affect behaviors by interfering with synaptic plasticity. In addition, internal and external stimuli could induce damage to mtDNA and mitochondrial dysfunction (36). Patients with severe depression usually demonstrate mitochondrial energy metabolism disorder, such as a lower level of ATP and a higher level of ROS, as well as the reduction of mitochondrial copy number (37). The results of our study suggested ZSQGY could attenuate the level of 8-OHdG and MDA, which are markers of oxidative stress during mitochondrial disturbance. In addition, ZSQGY could improve the decrease of ATP content and mtDNA copy number in the MSG-induced depression model. These findings indicate that ZSQGY could restore mitochondrial function.

It is well established that patients with depression exhibit increased circulating levels of inflammatory factors (38, 39). Inflammatory cytokines are key regulators of neuronal functions, and they play an important role in maintaining synaptic plasticity (40). Long-term exposure to psychosocial pressure disturbs the inflammatory cascade, leading to excess or prolonged inflammatory cytokines that interfere with synaptic plasticity and eventually leading to depressive symptoms (41, 42). Excessive ROS production and mtDNA damage induced by stress also trigger an inflammation response (43). Notably, ZSQGY was able to reduce the levels of IL-1β, IL-6, TNF-α, and IFN-γ, which demonstrated the anti-inflammatory effects of ZSQGY in the central nervous system in the MSG-induced depression model.

PGC-1α is a well-known ligand-activated transcription co-activator that mainly expresses in high energy demand tissues, e.g., brain, heart, and kidney (44). Once activated by different stimuli, PGC-1α translocates from the cytoplasm to the nucleus, where it interacts with nuclear respiratory factor 1 (NRF-1) and NRF-2 (45). As a result, mitochondrial transcription factor A (TFAM) is activated, increasing the expression of nuclear genes and further promoting mtDNA replication and transcription (46). It has been reported that PGC-1α deficiency could influence oxidative metabolism, then leading to axonal degeneration in the brain. PGC-1α also participates in the procedure of synaptogenesis. PGC-1α can enhance the expression of synaptic proteins, such as PSD95 (47, 48). In addition, PGC-1α is engaged in the process of macrophage polarization from the proinflammatory M1 phenotype to the anti-inflammatory M2 phenotype and then participates in the inflammation responses (49, 50). In this study, we found that ZSQGY could increase the expression of PGC-1α, accompanied by improvements in synaptic plasticity, mitochondrial function, and inflammation responses. Our findings are in line with the previous studies in which upregulating the level of PGC-1α could improve depression-like behaviors (51, 52). Moreover, the beneficial effects of ZSQGY were reversed after the administration of PGC-1α siRNA in the CORT-induced PC12 cell model. These results indicated that ZSQGY effectively could improve depressive behaviors *via* the mechanisms that regulate synaptic structural plasticity, improve mitochondrion function and alleviate neuroinflammation, which could, or partly, attribute to the regulation of PGC-1α.

The water extract of ZSQGY contains many bioactive compounds, however, in which chemical ingredients responsible for the beneficial effects of ZSQGY remain unknown. Here, we detected six major compounds of ZSQGY, including saikosaponin A, ferulic acid, albiflorin, paeoniflorin, geniposide, and ursolic acid. Previous studies have demonstrated that these chemical ingredients displayed anti-depressive effects in models of depression and the therapeutic mechanisms underlying these ingredients involve neuroendocrine, neuroinflammation, and neurotrophic systems (53). These findings provided evidence for the therapeutic effects of ZSQGY. In future, more studies are needed to discover the mechanisms that are underlying the synergistic antidepressant effects of these chemical ingredients.

## Data availability statement

The raw data supporting the conclusions of this article will be made available by the authors, without undue reservation.

## Ethics statement

The animal study was reviewed and approved by Institutional Ethics Committee of Zhongshan Hospital, Fudan University.

## Author contributions

WZhu and FY contributed to study design, data interpretation, and revision of the manuscript. WZha contributed to revision of the manuscript. MC and YX contributed to data interpretation. JX and XL contributed to statistical analysis and data interpretation. YY and DC provided final approval to submit the manuscript for publication. All authors reviewed the manuscript and approved the final manuscript.

## Funding

This work was supported by the Development Project of Shanghai Peak Disciplines-Integrative Medicine (No. 20180101).

## Conflict of interest

The authors declare that the research was conducted in the absence of any commercial or financial relationships that could be construed as a potential conflict of interest.

## Publisher’s note

All claims expressed in this article are solely those of the authors and do not necessarily represent those of their affiliated organizations, or those of the publisher, the editors and the reviewers. Any product that may be evaluated in this article, or claim that may be made by its manufacturer, is not guaranteed or endorsed by the publisher.
